# Factors associated with unplanned readmissions within 1 day of acute care discharge: a retrospective cohort study

**DOI:** 10.1186/s12913-018-3527-6

**Published:** 2018-09-14

**Authors:** Julie Considine, Debra Berry, Evan Newnham, Matthew Jiang, Karen Fox, David Plunkett, Melissa Mecner, Peteris Darzins, Mary O’Reilly

**Affiliations:** 1 Geelong: School of Nursing and Midwifery and Centre for Quality and Patient Safety – Eastern Health Partnership, Deakin University, Geelong, Australia; 2 Eastern Health, Box Hill, Australia; 3 Eastern Health Clinical School, Faculty of Medicine, Nursing and Health Sciences, Monash University, Melbourne, Australia

**Keywords:** Patient safety, Patient readmission, Hospital readmission, Discharge planning, Hospitalization, Health services

## Abstract

**Background:**

Unplanned hospital readmissions are a quality and safety indicator. In Australian, 8% to 11.1% of unplanned readmissions occur ≤1 day of acute care discharge. The aim of this study was to explore the reasons for unplanned hospital readmissions ≤1 day of acute care discharge, and determine what proportion of such unplanned hospital readmissions were potentially preventable.

**Methods:**

A retrospective exploratory cohort design was used to conduct this two phase study. In Phase 1, organisational data from 170 readmissions ≤1 day and 1358 readmissions between 2 and 28 days were compared using the Cochran-Mantel-Haenszel test. Binary logistic regression was used to examine factors associated with unplanned readmission ≤1 day. In Phase 2, a medical record audit of 162 Phase 1 readmissions ≤1 day was conducted and descriptive statistics used to summarise the study data. Index discharges occurred between 1 August and 31 December 2015.

**Results:**

In Phase 1, unplanned readmissions ≤1 day were *more likely* in paediatric patients (< 0.001); index discharges on weekends (*p* = 0.006), from short stay unit (SSU) (*p* < 0.001) or against health professional advice (*p* = 0.010); or when the readmission was for a Diagnosis Related Group (p < 0.001). The significant predictors of unplanned readmission ≤1 day were index discharge against advice or from SSU, and 1–5 hospital admissions in the 6 months preceding index admission.

In Phase 2, 88.3% readmissions were unpreventable and 11.7% were preventable. The median patient age was 57 years and comorbidities were uncommon (3.1%). Most patients (94.4%) lived at home and with others (78.9%). Friday was the most common day of index discharge (17.3%) and Saturday was the most common day of unplanned readmission (19.1%). The majority (94.4%) of readmissions were via the emergency department: 58.5% were for a like diagnosis and pain was the most common reason for readmission.

**Conclusions:**

Advanced age, significant comorbidities and social isolation did not feature in patients with an unplanned readmission ≤1 day. One quarter of patients were discharged on a Friday or weekend, one quarter of readmissions occurred on a weekend, and pain was the most common reason for readmission raising issues about access to services and weekend discharge planning.

## Background

In Australia, the demand for hospital care is increasing, [1] while average length of hospital stay (LOS) is decreasing, [1] resulting in increased patient acuity and turnover. Unplanned hospital readmissions within 28 or days of hospital discharge are commonly used indicators of quality and safety of healthcare. [2–4] Recent studies from Victoria and South Australia show 28 to 30-day unplanned readmission rates of 6.2 to 10.9%, [2, 5] [6] with the highest rates observed in adult medical patients. [6] However, Victorian data show that 11.1% of unplanned readmissions occurred within one day of discharge [6] and South Australian data show most likely day for readmission was on the first day (8%) after discharge. [5, 6] It may be proposed that unplanned hospital readmission within one day of discharge is an adverse events that indicates premature discharge or failure of discharge planning and that better understanding the characteristics and outcomes of patients readmitted within days of discharge can inform discharge planning.

## Methods

### Aim

There are no published studies focusing on unplanned hospital readmissions within the first days of discharge even though one in ten unplanned hospital readmissions occur within one day of discharge. Therefore, the aim of this study was to better understand the reasons for unplanned hospital readmissions within one day of acute care discharge, and determine what proportion of such unplanned hospital readmissions were potentially preventable.

### Design

This study was conducted in two phases using retrospective exploratory cohort design. In Phase 1, organisational data from 1528 unplanned readmissions within 28 days of acute care discharge was used to compare all cause readmissions occurring within 1 day to readmissions between two and 28 days following acute care discharge. In Phase 2, a medical record audit of readmissions within one day of acute care discharge was conducted. The study was approved by the Eastern Health Human Research Ethics Committee (LR22/2017) and a waiver of consent was granted.

### Setting

The study was conducted at Eastern Health, one of the largest health services in Victoria, Australia. Eastern Health serves a community a 750,000 people living across 2916 km^2^ and has Eastern Health has four acute care hospitals, three of which have emergency departments (EDs) and two of which have intensive care units (ICUs). Site A was an outer metropolitan hospital that provides emergency care, general medicine, surgery, midwifery, paediatrics and rehabilitation services. Site B was a tertiary referral centre providing all services except transplant surgery, neurosurgery and cardiothoracic surgery. Site C was a metropolitan hospital providing emergency care, general and specialist medicine, general and specialist surgery, critical care services and specialist adult mental health services, but no midwifery services. Site D was an outer metropolitan hospital providing medical, ambulatory and palliative care services. During 2014–15, there were 135,636 acute care admissions, 151,810 ED attendances and 31,083 operations performed at Eastern Health. [7]

There is some variability in the discharge process and discharge decision-making within the health service. In some units final authorisation for discharge is provided by consultant or registrar level medical staff and in other units, the Nurse Unit Manager authorise discharges. Discharge planning in some areas, for example, General Medicine, Rehabilitation Medicine, and Geriatric Medicine units is a highly structured, multi-disciplinary process. In other units, discharge decision-making can be less well defined, and is often principally a medical decision as distinct from a team decision. This difference reflects, in part, variability in the availability of allied health staff and the degree to which the patient journey is predictable.

### Sample

The sample for Phase 1 was 1528 unplanned readmissions ≤28 days in 1306 patients in whom the index discharge occurred between 1 August and 31 December 2015. Of these 1528 unplanned readmissions, 11.1% (*n* = 170) of readmissions occurred ≤1 day of acute care discharge: these patients were the sample for Phase 2. A re-admission was defined as a presentation resulting in admission to either a short stay unit (SSU) or inpatient ward.

### Data collection

The study data were extracted from organisational databases (Phase 1) and medical record review (Phase 2). In Phase 1, the following data were extracted from organisational databases: patient demographics, index admission characteristics, discharge planning, and reasons for readmission. For patients readmitted via the ED, triage category and ED LOS were also collected. International Classification of Diseases 10th revision Australian Modification (ICD-10-AM) codes [8] were used to calculate the Charlson Comorbidity Score: [9, 10] a score of 0 indicated no comorbidities. Complications were defined as ICD-10-AM codes with a ‘C’ prefix. [11] Organisational data is cleaned and checked by health information system staff before submission to the government. In Australia, data coding is closely monitored for compliance with coding standards because public hospital funding is dependent on accurate data coding. [12, 13] Data for Phase 2 were collected by two researchers (DB and MJ) using a detailed data dictionary, and inter-rater reliability was established using three cases that were independently audited by both researchers and reviewed by a chief investigator (MOR).

### Statistical analyses

Data were analysed using Statistical Package for Social Sciences (SPSS) Version 23.0. [14] Descriptive statistics were used to summarise the data; where data were not normally distributed, median and interquartile ranges (IQR) are presented. Patients were classified using Health Innovation and Reform Council definitions of obstetric patient, paediatric patient, adult surgical patient and adult medical patient [2, 6] Like DRGs were defined as the index admission and readmission diagnoses being from the same ICD-10 disease classification chapter heading. In Phase 1, patient and index admission characteristics (readmission within 1 day versus 2–28 days) were compared using the Cochran-Mantel-Haenszel (CMH) test to account for hospital clustering effects. Using the variables that were statistically significant, binary logistic regression was used to examine factors associated with unplanned readmission ≤1 day.

The determination of preventable readmission is subjective. A systematic review of 34 studies of avoidable readmissions showed that most studies used only one reviewer, relied on treating team and hospital factors, and gave little consideration to patient factors and social circumstances. [15] In Phase 2, we endeavoured to address these limitations by ensuring that social circumstances were examined in addition to system factors.

Readmissions were classified by the auditors (DB and MJ) as clearly unpreventable; requires further review; or clearly preventable. Clearly unpreventable readmissions were readmissions for issues unrelated to the index admission, and clearly preventable readmissions were those related to the index admission where the patient had signs of clinical instability or complex care needs on the day of index discharge, or where there had been a failure of discharge planning. For example, pain was a common reason for unplanned readmission, so the criteria for an unpreventable readmission for pain were that the patient was discharged on analgesia, last pain score documented ranged from 0 to 5 out of 10 and the patient did not require analgesia stronger than that prescribed for discharge on day of discharge. If a patient required intravenous, subcutaneous or oral opioid analgesia on the day of discharge but was discharged with simple oral analgesics, the readmission for pain was considered preventable.

The readmissions classified as requiring further review or clearly preventable were subject to a consensus process of further assessment of patient notes, vital signs, pathology and radiology reports, medication charts, social circumstances and discharge summary by three researchers (DB, an experienced registered nurse and nurse researcher; MOR, an infectious diseases physician and executive clinical director of ambulatory and community services with experience in audit of unplanned readmissions; and MJ, a medical registrar). Preventability was assessed qualitatively by file review with a specific focus on any factors that could have contributed to the readmission and that could have been addressed in the previous admission including at time of discharge, for example, unresolved factors related to the original admission diagnosis, unstable comorbidities, or documented evidence of carer burden or lack of social supports.

## Results

### Phase 1

Of the 1528 unplanned readmission occurring within 28 days of acute care discharge, 170 (11.1%) occurred within one day. The relationship between index admission and readmission characteristics for those readmitted within one day were compared to those readmitted within 2–28 days (Table 1).

**Table 1:** Patient and index admission characteristics: unplanned readmissions within 1 day versus 2–28 days

Patient and index admission characteristics	Discharges	Unplanned readmission ≤1 days (*n* = 170)		Unplanned readmission 2–28 days (*n* = 1358)		p
	n	n	%	n	%	
Male gender	685	82	48.2	755	44.4	0.307
Emergency admission^#^	1109	131	77.1	978	72.0	0.138
Chronic illness	800	81	47.6	719	52.9	0.224
Comorbidities (Charlson Index ≥1)	39	3	1.8	36	2.8	0.468
Patient group						
• Obstetric	79	9	5.3	70	5.2	0.944
• Paediatric	57	15	8.8	42	3.1	< 0.001
• Surgical	255	24	14.1	231	17.0	0.359
• Medical	1137	122	71.8	1015	74.7	0.427
Admission source						
• Home	1442	160	94.1	1282	94.4	0.889
• Nursing home	36	5	2.9	31	2.3	0.604
• Hospital transfer	45	4	2.4	41	3.0	0.631
Complications during index admission	1155	133	78.2	1022	75.3	0.417
Hospital admissions in last 6 months	773	103	60.6	663	48.8	0.005
ED attendances in last 6 months	752	71	41.8	681	50.1	0.047
Readmission - like DRG	724	101	59.4	623	45.9	0.001
Index LOS greater than State average for DRG	389	35	20.6	354	26.1	0.135
Discharge at weekend	300	47	27.6	253	18.6	0.006
Index admission - SSU	366	61	35.9	305	22.5	< 0.001
Index admission - AMU	305	26	8.5	279	20.5	0.104
Discharge destination						
• Transitional care	6	2	1.2	4	0.3	0.107
• Left against advice	20	6	3.5	14	1.0	0.010
• Private home	1336	149	87.6	1187	87.4	0.936
• Aged care	49	5	3.9	44	3.2	0.820
Age						
• 0 to 17 years	58	15	8.8	43	3.2	0.001
• 18 to 64 years	697	87	51.2	610	44.9	0.134
• 65 to 84 years	566	53	31.2	513	37.8	0.104
• 85 and over	207	15	8.8	192	14.1	0.058

*ED* emergency department; *DRG* diagnostic related group; *LOS* length of stay; *SSU* short stay unit; *AMU* acute medical unit

Binary logistic regression was performed to compare unplanned readmissions within 1 day and 2–28 days, using the variables that were statistically significantly associated with increased risk of readmission within 1 day in the bivariate analysis (Table 1). The results of multivariate analyses are presented in Table 2. A test of the full model against constant only model was statistically reliable (omnibus χ^2^ = 52.485, d.f. = 9, *p* < 0.001 and Hosmer Lemeshow test *p* = 0.930), and the model correctly classified 88.9% of cases. When adjusted for age and site, the significant predictors of unplanned readmission within 1 day were index discharge against advice, index admission under short stay unit and 1–5 hospital admissions in the 6 months preceding the index admission (Table 2).

**Table 2:** Factors associated with increased risk of unplanned readmissions within 1 day of acute care discharge

	OR	95% CI	p
Index discharge against advice	3.805	1.405–10.302	0.009
Index admitting unit SSU	1.788	1.254–2.550	0.001
Hospital admissions in 6 months preceding index admission			
• Nil (reference)			
• 1–5	0.665	0.465–0.951	0.025
• 6–10	0.327	0.100–1.070	0.064
• > 10	1.972	0.936–4.156	0.074
Index discharge on weekend	1.421	0.977–2.067	0.066
Age	0.988	0.981–0.994	< 0.001
Health service site			
• Site A (reference)			
• Site B	1.002	0.598–1.680	0.993
• Site C	0.965	0.551–1.690	0.900

*OR* odds ratio; *CI* confidence interval; *SSU* short stay unit

### Phase 2

Of the 170 discharges included in Phase 2, seven (4.1%) were excluded; planned admission for induction of labour (*n* = 1), planned surgery (*n* = 3), iron infusion (*n* = 1), removal of nasal tamponade (*n* = 1), no available notes for readmission (*n* = 1), and readmission from Hospital in the Home (HITH)). The remaining 162 readmissions occurred in 160 patients: two patients had two discharges resulting in unplanned readmission ≤1 day during the period studied. After medical record review, there were 138 (85.2%) unpreventable readmissions, 5 (3.1%) readmissions that underwent further review and were deemed by the consensus panel to be unpreventable, and 19 readmissions (11.7%) that were confirmed as preventable by the consensus panel.

When all readmissions within 1 day were examined, the median patient age was 57 years (IQR = 32 to 77), 81 (50%) were male, 119 (73.5%) were adult medical patients, 24 (14.8%) were adult surgical patients, and the majority (*n* = 147, 90.7%) had a preferred language of English. Co-morbidities were present in 5 patients (3.1%). Of the 19 preventable readmissions, the median patient age was 68 years (IQR =44–82), 13 (68.5%) were female, 15 (78.9%) were adult medical patients, and only one patient had known comorbidities. Most patients with preventable readmissions within 1 day (*n* = 16, 84.2%) had a preferred language of English and lived with others (*n* = 15, 78.9%). The patient characteristics of all readmissions within 1 day and preventable readmissions within 1 day are presented in Table 3.

**Table 3:** Patient characteristics: all versus preventable unplanned readmissions within 1 day

	All unplanned readmissions ≤1 day (*n* = 162)		Preventable unplanned readmissions ≤1 day (*n* = 19)	
	n	%	n	%
Patient group				
• Paediatric	12	7.4	1	5.2
• Obstetric	7	4.3	0	0.0
• Adult Surgical	24	14.8	3	15.7
• Adult Medical	119	73.5	15	78.9
English as preferred language	147	90.7	16	84.2
Charlson Comorbidity Score				
• 0	157	96.9	17	89.5
• 1	3	1.9	1	5.3
• 2	2	1.2	1	5.3
• 3	0	0	0	0.0
• ≥4	0	0	0	0.0
Patients’ living arrangements				
• With others	127	78.9	15	78.9
• On own	20	12.4	3	15.8
• Supported living	8	5.0	0	0.0
• Residential aged care	6	3.7	1	5.3
Admission source				
• Home	153	94.4	18	94.7
• Residential Aged Care	5	3.1	1	5.3
• Other Hospital / Extended Care / Rehabilitation	4	2.5	0	0.0

### Index admission characteristics

The index admission characteristics of all readmissions and preventable readmissions within 1 day are presented in Table 4. During the index admissions, there were no Medical Emergency Team (MET) activations and three Intensive Care Unit (ICU) admissions.

**Table 4:** Index admission characteristics: all versus preventable unplanned readmissions within 1 day

	All unplanned readmissions ≤1 day (*n* = 162)		Preventable unplanned readmissions ≤1 day (*n* = 19)	
	n	%	n	%
Emergency admission^*^	126	77.8	17	89.5
Admitting Program				
• Emergency Medicine^**^	60	37.0	12	63.2
• Surgical	39	24.1	2	10.5
• General Medical	26	16.0	3	15.7
• Specialty Medicine	21	13.0	0	0.0
• Women’s Health	11	6.8	1	5.3
• Children’s	5	3.1	1	5.3
Admitted as a boarder to a ward not related to their speciality	15	9.3	1	5.3
Complications during index admission				
• Bleeding/haemorrhage	4	2.5	0	0.0
• Cardiac	4	2.5	1	5.3
• Respiratory	4	2.5	0	0.0
• Abnormal laboratory test results	4	2.5	0	0.0
• Urinary Retention	3	1.8	0	0.0
• Fever/Sepsis	3	1.8	0	0.0
• Altered Conscious State	2	1.2	0	0.0
• Acute Kidney Injury	2	1.2	0	0.0
• Hypotension	2	1.2	0	0.0
• Pain	2	1.2	0	0.0
• Anxiety	1	0.6	0	0.0
• Cancer	1	0.6	0	0.0
• Pseudo seizure	1	0.6	0	0.0
• Malnutrition	1	0.6	1	5.3

^*^Emergency admission refers to an unplanned hospital admission via the emergency department (opposed to an elective admission): these patients can be admitted under any admitting program

^**^Admitting program ‘Emergency’ refers to patients admitted to short stay units under the emergency medicine program

Diagnostic uncertainty was present in 18 (11.1%) of all index discharges. Only 1 discharge resulting in a preventable unplanned readmission within 1 day involved diagnostic uncertainty. Index admissions with unresolved primary problems included those in which the patient discharged themselves against medical advice, had planned definitive treatments but with current symptoms controlled (e.g. requiring surgical interventions), or those with no clear discharge diagnosis. The patient’s primary problem was unresolved in 51 (31.5%) of all index admissions and 11 (57.9%) index admissions associated with a preventable unplanned readmission within 1 day. For the whole cohort, 126 (77.8%) index admissions occurred via the ED, 93 (76.2%) patients were triaged as requiring emergency care within 30 min, and the median ED length of stay was 5.3 h (IQR = 3.6 to 9.9 h). For patients whose readmission was preventable, 17 (89.5%) of index admissions were via the ED, 11 (57.9%) patients were triaged as requiring emergency care within 30 min, and the median ED length of stay was 5.3 h (IQR = 3.8 to 9.1 h).

In the six months preceding the index admission, 69 (42.6%) patients had one or more ED attendances and 66 (40.7%) patients had one or more hospital admissions. One third of patients (*n* = 55, 33.9%) had *both* ED attendance(s) and hospital admission(s) and 82 patients (50.6%) patients had neither an ED attendance nor a hospital admission. The median hospital length of stay for index admissions was 1 day (IQR = 1 to 3 days). Of the patients who had a preventable unplanned readmission, 11 (57.9%) patients had one or more ED attendances and 11 (57.9%) patients had one or more 1 hospital admissions. ED attendance(s) *and* hospital admission(s) occurred in 7 (36.8%) patients and 10 (52.6%) patients had neither an ED attendance nor a hospital admission. The median hospital length of stay for index admissions associated with a preventable unplanned readmission was 1 day.

### Index admission discharge planning

For all patients with an unplanned readmission within 1 day of acute care discharge, Friday was the most common day of discharge (*n* = 28, 17.3%) and 43 (26.5%) discharges occurred on a weekend. In most instances, the patient went home (*n* = 141, 87.0%) and to an environment where they lived with others (*n* = 127, 78.9%). The majority of discharges resulted in a referral to outpatients (*n* = 67, 41.1%) but in 37 (22.8%) discharges no formal discharge referrals were made.

For patients in whom the unplanned readmission was preventable, Sunday was the most common day of index discharge (*n* = 6, 31.6%) and 7 (36.8%) discharges occurred on a weekend. Again, in most instances, the patient went home (*n* = 17, 89.5%) and to a setting where they lived with others (*n* = 15, 78.9%). An outpatients referral was made in almost half of these patients (*n* = 9, 47.4%). The index discharge destination and referrals for all readmissions and preventable readmissions within 1 day of acute care discharge are presented in Table 5.

**Table 5:** Index discharge destination and referrals: all versus preventable unplanned readmissions within 1 day

	All unplanned readmissions ≤1 day (*n* = 162)		Preventable unplanned readmissions ≤1 day (*n* = 19)	
	n	%	n	%
Discharge destination				
• Home	141	87.1	17	89.5
• Against medical advice	6	3.7	0	0.0
• Residential aged care	5	3.1	1	5.3
• Transitional care program	2	1.2	0	0.0
Discharge referrals				
• Outpatients	67	41.1	9	47.4
• None	37	22.8	2	10.5
• General Practitioner	29	17.9	5	26.3
• Midwifery/obstetrics	8	4.9	0	0.0
• Specialist follow-up	8	4.9	2	10.5
• Other hospital	6	3.7	0	0.0
• Community services (meals on wheels, community or district nursing)	3	1.9	1	5.3
• Care transferred to hospital in the home	2	1.2	0	0.0
• Hospital admission risk program (HARP)	1	0.6	0	0.0

### Characteristics of unplanned readmissions within 1 day

For the entire cohort, most common day of unplanned readmission within 1 day was Saturday (*n* = 31, 19.1%) and 45 (27.7%) readmissions occurred on a weekend. One third of readmissions (*n* = 53, 32.7%) occurred overnight (2200–0759 h) and in two thirds of readmissions (*n* = 110, 68.3%) the patient made the decision to return to hospital. The majority (*n* = 153, 94.4%) of readmissions were via the ED and transport to hospital by ambulance occurred in 59 (40.7%) of readmissions. The majority of patients (*n* = 121, 80.7%) were triaged as warranting emergency care within 30 min. The reason for readmission was for a like DRG in 95 (58.6%) readmissions, 141 (88.7%) readmissions were for a problem related to the index admission problem, and pain was the most common reason for readmission (Table 6).

**Table 6:** Readmission characteristics

	All unplanned readmissions ≤1 day (*n* = 162)		Preventable unplanned readmissions ≤1 day (*n* = 19)	
	n	%		
Mode of arrival to hospital				
• Ambulance	59	40.7	6	31.6
• Private car	86	59.3	0	0.0
• Police	1	0.7	11	57.9
Readmission referral				
• Self	110	68.3	13	68.4
• GP	2	1.2	N/A	N/A
• Specialist	4	2.5	1	5.3
• Emergency Department	3	1.9	N/A	N/A
• Residence (RAC/SRS)	9	5.6	1	5.3
• Other*	33	20.5	4	21.0
Reason for readmission				
• Pain	47	29.2	5	26.3
• Other	21	13.0	0	0.0
• Shortness of breath	14	8.7	3	15.7
• Bleeding	14	8.7	1	5.3
• Infection (not wound)	14	8.7	0	0.0
• Wound issues / infection	9	5.6	2	10.5
• Falls	7	4.3	2	10.5
• Nausea/Vomiting	7	4.3	1	5.3
• Collapse	5	3.1	1	5.3
• Labour	4	2.5	0	0.0
• Confusion/behaviour	4	2.5	2	10.5
• Medication adverse effects	3	1.9	0	0.0
• Pneumonia	3	1.9	0	0.0
• Seizure	3	1.9	0	0.0
• Urinary retention	3	1.9	0	0.0
• Hip Dislocation	3	1.9	0	0.0
• Overdose	0	0.0	1	5.3
• Fever	0	0.0	1	5.3

* Includes but not limited to parents, adult children and partners

For patients who experienced a preventable readmission within 1 day, the most common day of unplanned readmission within 1 day was Monday (*n* = 7, 36.8%) and only 2 readmissions occurred on a weekend. One third of readmissions (*n* = 7, 36.8%) occurred overnight (2200–0759 h) and in two thirds of readmissions (*n* = 13, 68.4%), the patient made the decision to return to hospital. All (*n* = 19) preventable readmissions within 1 day were via the ED and ambulance transport occurred in 6 (31.6%) cases. Thirteen (68.4%) patients were triaged as warranting emergency care within 30 min. The reason for readmission was for a like DRG in 9 (47.4%) preventable readmissions, 18 (94.7%) readmissions were for a problem related to the index admission problem, and again pain was the most common reason for readmission (Table 6). The readmission characteristics for all readmissions within 1 day and preventable readmissions within 1 day are presented in Table 6.

In 13 (8.0%) unplanned readmissions within 1 day, there was one or more MET activations during that admission and the median time to first MET call was 30 h (IQR = 12 to 107). There were one or more ICU admissions in 5 (3.1%) readmissions and the median time to first ICU admission was 7 h (IQR = 0 to 57). In three readmissions (1.9%), the patient died in hospital during that readmission. Two patients (10.6%) who experienced a preventable unplanned readmission ≤1 day had a MET all during that admission, and the median time to MET activation was 75 h. There were no ICU admissions or in-hospital deaths in the preventable readmission group. The median hospital length of stay during the readmission was 2 days (IQR = 1 to 4) for all patients readmitted within 1 day and 1 day (IQR = 1 to 4) for patients whose unplanned readmission was preventable.

## Discussion

This study had four major findings. First, the major predictors of unplanned readmission ≤1 days were index discharge against advice, and index admission and discharge under the SSU. Second, advanced age, significant comorbidities and social isolation did not feature in patients who experienced unplanned readmission within 1 day nor do they seem to have an association with preventable unplanned readmissions, however, almost half the patients studied used healthcare services (ED attendance and, or hospital admission) in the six months preceding the index admission. Third, unplanned readmissions within 1 day were commonly associated with reason for index admission and pain was the most common reason for all unplanned readmissions and preventable readmissions within 1 day. Finally, the most common day of readmission was Saturday and just under one third of readmissions within 1 day occurred on the weekend.

One quarter of index discharges resulting in an unplanned readmission within 1 day were from the SSU (27.8%) which may, in part, explain the short median hospital length of stay for index admissions of 1 day. The most common admitting ward for preventable readmissions was also SSU. These findings may in part be explained by the intent and nature of SSU care. The purpose of SSUs is to continue to provide care to ED patients who require a period of observation or therapy [16] therefore an element of diagnostic uncertainty that may or not be resolved during SSU care is plausible. For some patients, symptom resolution often results in a desire to go home. Inherent in supporting the patient’s choice to go home is the risk of symptom recurrence or condition deterioration resulting in an unplanned ED attendance and, or hospital readmission. SSUs are different to the rest of the hospital with much higher patient turnover. For example, at the study site there were 2801 SSU admissions during June 2017 compared with 1057 admissions to general medical units. Other studies of outcomes of SSU care report ED representation rates of 3.9% to 6.2% within the first week after discharge from SSU [17] and in cardiac patients, hospital readmission rates of 6.1% within 8 weeks [18] and 8% within 90 days [19] of SSU discharge.

One in ten unplanned hospital readmissions within 28 days of acute care discharge occurred within 1 day. This finding is consistent with other Australian studies that have shown the most likely day for unplanned hospital readmission was on the first day discharge and that 8–9% of unplanned readmissions occurred within 1 day. [5, 20] In our study 11.7% of unplanned readmissions within 1 day of acute care discharge were deemed preventable. The proportion of preventable unplanned readmissions reported in the literature ranges from 5 to 79% (median of 27.1%). [15] This variation is likely due to inconsistency in definitions of preventable and difficulties in reliability determining preventability. The 19 preventable unplanned readmissions examined in this study were drawn from a total of 20,575 discharges so less than 0.1% of index discharges result in a preventable unplanned readmission within 1 day. [6] A study of 4812 admissions showed preventable unplanned readmission (at any point in time) was 2.2% (IQR 1.5–7.0%). [21] A systematic review of 37 studies showed that although there was significant variation in index conditions, readmission conditions, timeframes examined and terminology used, the common patient factors associated with preventable hospital readmission were indicators of poor health or frailty such as co-morbidities, illness severity, general poor health or high use of the healthcare system. [22]

Our audit data showed that unplanned readmissions within 1 day occurred in middle aged patients (median = 57 years), most of whom lived at home (94.4%), preferred to speak English (90.7%) and were largely comorbidity free (97.5%). Patients in whom the unplanned readmission was judged as preventable were slightly older (median age = 68 years), more likely to be female (68.5%), commonly lived with others (78.9%) and only one patient had known comorbidities. These findings suggest that advanced age, significant comorbidities and social isolation do not appear to feature in our patients who experience unplanned readmission within 1 day nor do they seem to have an association with preventable unplanned readmissions. Although not focused specifically on unplanned readmissions ≤1 day, other studies of unplanned readmissions have also shown that both preventable and non-preventable unplanned readmissions tend to occur in middle-aged patients, most of whom were living independently without the need for support of activities of daily living and free of serious comorbidities. [23] The health, wellbeing and capability of at-home carers may be an area for further research and the reason for the increased proportion of females in our preventable readmissions group is unclear.

The reason for readmission was for a like DRG in two thirds of readmissions and 88.7% of readmissions were for a problem related to the reason index admission. Other studies report that 44% to 71% of patients are admitted for the same reason as their index admission. [24, 25] Diagnostic uncertainty was present in one in ten index discharges but there was considerable variation and no major source of diagnostic uncertainty. The primary problem remained unresolved in one third of index admissions. There is limited reporting of diagnostic uncertainty as a factor in unplanned readmissions. The literature to date has focused on diagnostic error, inadequate assessment or failures to order, interpret or follow up diagnostic tests. [26] Pain was the most common reason for readmission, occurring in almost one third of readmissions. There are few published studies that show that although pain is commonly a factor in unplanned readmissions, [27–29] pain score on discharge is not a useful predictor of readmission. [27] However, pain score at discharge in combination with analgesia use and age ≥ 75 years has been shown to be a risk factor for the number of unplanned readmissions within 30-days. [27]

One quarter of all unplanned readmissions ≤1 day experienced a complication during their index admission (22.7%) however there was variability in the types of complications that occurred and no dominant complication theme. The incidence of clinical deterioration during index admission (MET calls or ICU admissions) was also low. There is evidence that clinical deterioration during index hospitalisation increase the risk of unplanned readmissions [30] however the specifics of these adverse events are poorly defined in the literature. Health service use in the six months preceding the index admission was evident in approximately half of patients; 42.6% of patients had ≥1 ED attendances and 40.7% of patients had ≥1 hospital admissions. Increased health service use is a known risk factor for unplanned hospital readmissions at 28 to 90 days [26, 30] but this is the first study to examine health service use preceding unplanned readmission within 1 day. The degree of health service use seems a disparate finding our patients who were not particularly old and had few comorbidities. Comorbidities were measured in this study using the Charlson Comorbidity Score. These findings raise questions about whether the Charlson Comorbidity Score captures all the conditions such as pain states, cognition, frailty that may result in rehospitalisation to the degree necessary to plan care. Further, there is some evidence that in medically complex patients, functional status is a better predictor of acute readmission than comorbidities. [31]

The most common day of index discharge was Friday which explains why Saturday was the most common day of unplanned readmission. Further, 26.5% of discharges occurred on a weekend again explaining why 27.7% of readmissions occurred on a weekend. These findings may reflect lack of access to alternative care providers, support services or health advice over the weekends. These findings are different to those of other Australian studies. In a study of readmissions to an Australian regional hospital, McLean [20] found that Fridays had the highest and weekends had the lowest number of unplanned readmissions. Li et al. [5] found that discharge on a weekend versus a week day did not affect the risk of unplanned readmission within 28-days in medical patients from major South Australian health service. The majority (94.4%) of unplanned readmissions were via the ED, in 40.7% of readmissions transport to hospital was by ambulance and the majority of patients (80.7%) returning via the ED were triaged as requiring emergency care within 30 min. These results suggest that although unplanned readmissions are increasing the workload of emergency care providers, many patients appeared to have an issue of considerable clinical urgency that may not have been amenable to management by other healthcare providers. Further, even if care by alternative providers was a clinical option, the results discussed previously suggest need for care on weekends was not uncommon raising issues of access to services. One in ten unplanned readmissions involved a MET call and the median length of hospital stay following readmission was 2 days which further supports that these patients were unwell.

There are a number of limitations that should be considered when interpreting the study findings. First, this work was retrospective and thus inherently reliant on medical record data. Therefore there may be patient and system factors that are not reflected in these data sources. The disparate results of low Charlson Comorbidity Scores and moderate degree of health service use in the 6 months preceding readmission, and reports that patient characteristics not captured in organisational or medical record data such as behaviours, that may influence readmission [32] add weight to this assertion. Further, organisational and medical record data do not provide detailed information about health care provider characteristics and there is emerging evidence of hospital factors that contribute to readmissions independent of patient factors. [33] Second, the study was conducted at a single health service so the generalisability of the study findings to other organisations may be limited. Third, our definition of readmission was readmission back to the same health service, it is possible that some discharges resulted in readmissions to other health services therefore the true readmission rate is under-reported. Finally, in the absence of a clear definition of preventability, determination of preventability was achieved using criteria specific to this study and a consensus process.

## Conclusion

When adjusted for age and site, the significant predictors of unplanned readmission ≤1 days were index discharge against advice, and index admission under short stay unit. Preventable unplanned readmission ≤1 day are uncommon however one in eight unplanned readmissions ≤1 day were deemed preventable. Advanced age, significant comorbidities and social isolation do not appear to feature in our patients who experience unplanned readmission ≤1 day, irrespective of whether those readmissions were preventable or non-preventable so challenges the notion that unplanned readmissions occur in predominantly older people with significant comorbidities and low levels of social support. Pain was the most common reason for readmission therefore pain management strategies on discharge and early follow up of patients discharged with a painful condition should be an area for further investigation. SSU was the clinical area associated with the highest number of overall and preventable unplanned readmissions ≤1 day, which may in part be explained by the high levels of patient turnover, but admission and discharge criteria for SSU, and follow up of patients discharged home from SSU may warrant further review. One quarter of patients experiencing unplanned readmissions ≤1 day were discharged on a Friday or weekend, and one quarter of readmissions occurred on a weekend raising issues about access to services and weekend discharge planning. Understanding whether there is a difference in discharge processes and how patients access or reconnect with services on weekends compared to weekdays should be a focus of future work and may inform a more consistent approach to service delivery.

## Abbreviations

CHM: Cochran-Mantel-Haenszel
DRG: Diagnostic related group
ED: Emergency department
ICD-10-AM: International Classification of Diseases 10th revision Australian Modification
IQR: Interquartile range
LOS: Length of Stay
SPSS: Statistical Package for Social Sciences
SSU: Short Stay Unit
AMU: Acute Medical Unit
OR: Odds ratio
CI: Confidence interval
MET: Medical Emergency Team
ICU: Intensive Care Unit

## References

[1] Australian Institute of Health and Welfare: Admitted patient care 2014–15: Australian hospital statistics. In*.* Canberra: Australian Institute of Health and Welfare. Health services series no. 68. Cat. no. HSE 172. Retrieved 6 September 2016 from https://www.aihw.gov.au/reports/hospitals/ahs-2014-15-admitted-patient-care/contents/table-of-contents; 2016. Accessed 9 Sept 2018.

[2] Health Innovation and Reform Council: Presenting the data considered in preparation of advice regarding improvement to unplanned readmission performance in Victoria. In*.* Melbourne, Victoria: Victorian Government, Department of Health; 2013.

[3] Gruneir A, Dhalla IA, van Walraven C, Fischer HD, Camacacho X, Rochon PA, Anderson GM: Unplanned readmissions after hospital discharge among patients identified as being at high risk for readmission using a validated predictive algorithm. Open Med 2011; 5:104–111. PMC3148002 21915234

[4] Zhou Huaqiong, Della Phillip R, Roberts Pamela, Goh Louise, Dhaliwal Satvinder S (2016). Utility of models to predict 28-day or 30-day unplanned hospital readmissions: an updated systematic review. BMJ Open.

[5] Li JYZ, Yong TY, Hakendorf P, Ben-Tovim DI, Thompson CH (2015). Identifying risk factors and patterns for unplanned readmission to a general medical service. Aust Health Rev.

[6] Considine J, Fox K, Plunkett D, Mecner M, O'Reilly M, Darzins P: Factors associated with unplanned readmissions in a major Australian health service. Aust Health Rev in press:accepted 28 September 2017. available on line 2011 November 2017. 10.1071/AH16287 29092726

[7] Quick Facts about Eastern Health [https://www.easternhealth.org.au/images/about/EH_Quick_Facts.pdf]. Accessed 9 Sept 2018.

[8] Gabbe BJ, Harrison JE, Lyons RA, Edwards ER, Cameron PA: Comparison of measures of comorbidity for predicting disability 12-months post-injury. BMC Health Ser Res 2013; 13:Published online 26 January 2013. 10.1186/1472-6963-13-30 PMC3562274 23351376

[9] Quan H, Sundararajan V, Halfon P, Fong A, Burnand B, Luthi J-C, Saunders LD, Beck CA, Feasby TE, Ghali WA (2005). Coding algorithms for defining comorbidities in ICD-9-CM and ICD-10 administrative data. Med Care.

[10] Charlson ME, Pompei P, Ales KL, MacKenzie CR (1987). A new method of classifying prognostic comorbidity in longitudinal studies: development and validation. J Chronic Dis.

[11] Santamaria JD, Tobin AE, Anstey MH, Smith RJ, Reid DA (2014). Do outlier inpatients experience more emergency calls in hospital? An observational cohort study. Med J Aust.

[12] Henderson T, Shepheard J, Sundararajan V: Quality of diagnosis and procedure coding in ICD-10 administrative data. 2006; 44:1011–1019. 10.1097/01.mlr.0000228018.48783.34 17063133

[13] Australian Refined Diagnosis Related Group (AR-DRG) classification system [https://www.ihpa.gov.au/what-we-do/products/AR-DRG-classification-system]. Accessed 9 Sept 2018.

[14] IBM Corporation: IBM SPSS (statistical package for social sciences) statistics version 23.0. In*.*, edn. Armonk, NY: IBM Corporation; 2015.

[15] Van Walraven C, Bennett C, Jennings A, Austin PC, Forster AJ: Proportion of hospital readmissions deemed avoidable: a systematic review. Can Med Assoc J 2011:cmaj. 101860. 10.1503/cmaj.101860 PMC3080556 21444623

[16] Daly S, Campbell DA, Cameron PA (2003). Short-stay units and observation medicine: a systematic review. Med J Aust.

[17] Juan A, Salazar A, Alvarez A, Perez JR, Garcia L, Corbella X (2006). Effectiveness and safety of an emergency department short-stay unit as an alternative to standard inpatient hospitalisation. Emerg Med J.

[18] Roberts RR, Zalenski RJ, Mensah EK, Rydman RJ, Ciavarella G, Gussow L, Das K, Kampe LM, Dickover B, McDermott MF (1997). Costs of an emergency department-based accelerated diagnostic protocol vs hospitalization in patients with chest pain: a randomized controlled trial. JAMA.

[19] Miller CD, Case LD, Little WC, Mahler SA, Burke GL, Harper EN, Lefebvre C, Hiestand B, Hoekstra JW, Hamilton CA (2013). Stress CMR reduces revascularization, hospital readmission, and recurrent cardiac testing in intermediate-risk patients with acute chest pain. JACC Cardiovascular imaging.

[20] McLean R, Mendis K, Canalese J (2008). A ten-year retrospective study of unplanned hospital readmissions to a regional Australian hospital. Aust Health Rev.

[21] Van Walraven C, Forster AJ (2013). When projecting required effectiveness of interventions for hospital readmission reduction, the percentage that is potentially avoidable must be considered. J Clin Epidemiol.

[22] Vest JR, Gamm LD, Oxford BA, Gonzalez MI, Slawson KM (2010). Determinants of preventable readmissions in the United States: a systematic review. Implement Sci.

[23] van Walraven C, Jennings A, Taljaard M, Dhalla I, English S, Mulpuru S, Blecker S, Forster AJ (2011). Incidence of potentially avoidable urgent readmissions and their relation to all-cause urgent readmissions. Can Med Assoc J.

[24] Magdelijns FJH, Schepers L, Pijpers E, Stehouwer CDA, Stassen PM (2016). Unplanned readmissions in younger and older adult patients: the role of healthcare-related adverse events. Eur J Med Res.

[25] Allaudeen N, Schnipper JL, John Orav E, Wachter RM, Vidyarthi AR (2011). Inability of providers to predict unplanned readmissions. J Gen Intern Med.

[26] Scott IA, Shohag H, Ahmed M (2014). Quality of care factors associated with unplanned readmissions of older medical patients: a case-control study. Intern Med J.

[27] Deschepper M, Vermeir P, Vogelaers D, Devulder J, Eeckloo K (2017). Is pain at discharge a risk factor for unplanned hospital readmission?. Acta Clin Belg Int J Clin Lab Med.

[28] Deschodt M, Devriendt E, Sabbe M, Knockaert D, Deboutte P, Boonen S, Flamaing J, Milisen K (2015). Characteristics of older adults admitted to the emergency department (ED) and their risk factors for ED readmission based on comprehensive geriatric assessment: a prospective cohort study. BMC Geriatr.

[29] Neuman MD, Wirtalla C, Werner RM (2014). Association between skilled nursing facility quality indicators and hospital readmissions. JAMA.

[30] Franchi C, Nobili A, Mari D, Tettamanti M, Djade CD, Pasina L, Salerno F, Corrao S, Marengoni A, Iorio A (2013). Risk factors for hospital readmission of elderly patients. Eur J Int Med.

[31] Shih SL, Gerrard P, Goldstein R, Mix J, Ryan CM, Niewczyk P, Kazis L, Hefner J, Ackerly DC, Zafonte R (2015). Functional status outperforms comorbidities in predicting acute care readmissions in medically complex patients. J Gen Int Med.

[32] Steventon A, Billings J (2017). Preventing hospital readmissions: the importance of considering 'impactibility,' not just predicted risk. BMJ Qual Saf.

[33] Krumholz HM, Wang K, Lin Z, Dharmarajan K, Horwitz LI, Ross JS, Drye EE, Bernheim SM, Normand S-LT (2017). Hospital-readmission risk — isolating hospital effects from patient effects. N Engl J Med.

